# Full-text publication of abstracts in emergency medicine in Denmark

**DOI:** 10.1186/1757-7241-22-33

**Published:** 2014-05-24

**Authors:** Anne Katrine Ravn, Dan Brun Petersen, Lars Folkestad, Peter Hallas, Mikkel Brabrand

**Affiliations:** 1 Emergency Department, Sydvestjysk Sygehus Esbjerg, Finsensgade 35, DK-6700 Esbjerg, Denmark; 2 Emergency Department, Holbaek Hospital, Smedelundsgade 60, 4300 Holbæk, Denmark; 3 Emergency Department & Department of Endocrinology, Sydvestjysk Sygehus Esbjerg, Finsensgade 35, DK-6700 Esbjerg, Denmark; 4 Department of Pediatric Anesthesia, 4013, Copenhagen University Hospital, Rigshospitalet, Blegdamsvej 3, 2100 Copenhagen, Denmark; 5 Department of Medicine, Sydvestjysk Sygehus Esbjerg, Finsensgade 35, DK-6700 Esbjerg, Denmark

## Abstract

**Introduction:**

Abstracts presented at medical conferences or scientific meetings should ideally be published as full-text articles in peer-reviewed journals after initial presentation and feedback regardless of the findings. The aim of this survey was to determine the publication rate of papers presented at the Danish Emergency Medicine Conferences in 2009, 2010 and 2011.

**Methods:**

Abstracts presented at the conferences were identified and authors contacted to obtain publication information. A further search was conducted using relevant databases.

**Results:**

Publication rates for the 2009 and 2010 were approximately 30% (25–31.6%). The publication rate for the 2011 conference was 14.5% within 18 months with an additional 9% under review prior to publication.

**Discussion:**

When comparing full-text publication rates from DEMC to previous international studies in EM Danish EM research community has similar publication rates. However, other more established specialties have higher publication levels. Knowledge of reasons for non-publication could lead to efforts to promote publication like funding; the possibility of discussion between authors and editors at conferences; “publication mentors”; and/or research courses provided by the Danish Society of Emergency Medicine.

## Introduction

Many researchers present their initial findings as abstracts at medical conferences or scientific meetings. These abstracts should ideally be published as full text articles in peer-reviewed journals after initial presentation and feedback regardless of the findings. Previous studies suggest that the rate of publication is subject to fluctuations when looking across specialties, often around or below 50% [1,2] and it seems to be a stable phenomenon over time [3,4]. Little is known about why some abstracts are not published as full-text articles at a later time, but insufficient priority or lack of time, funds or other resources have been suggested as reasons [5]. The publication rate of conference abstracts in Emergency Medicine (EM) internationally is approximately 30% [6,7].

EM is new in Denmark. However, the Danish Society for Emergency Medicine has arranged five conferences to date. The first two conferences in 2007 and 2008 were small and only a few abstracts were submitted. However, from the 2009 conference onwards, the number of accepted abstracts increased and publication of abstracts in an international scientific journal was arranged. Knowledge on the publication rates from the Danish conferences would help establish if initiatives are needed to improve publication rates and will provide a baseline for evaluation of such initiatives. We therefore performed the present survey with the aim of determining the publication rate of papers presented at the Danish Emergency Medicine Conferences (DEMC) in 2009, 2010, and 2011.

## Methods

Abstracts presented at DEMC in 2009, 2010 and 2011 were published as supplements in the Scandinavian Journal of Trauma, Resuscitation and Emergency Medicine (SJTREM) [8-10] after the conferences. In this cross-sectional study, we identified the abstracts and initially contacted the corresponding author of all published abstracts by e-mail during May 2013 with a request for information regarding:

1. If the data had been published?

2. If so, in full or in part?

3. Where was it published (reference)?

4. If not published yet, are some/all of the data scheduled for publication?

If we did not receive an answer to the first email, the corresponding author was contacted again after one week with a request to submit information.

For abstracts to which the corresponding author did not reply after the second email a manual search was conducted in PubMed and EMBASE (and CINAHL when appropriate) using names of authors and title keywords. Methodology, sample size and results were compared to ensure fit.

Abstracts submitted to the conferences competed to be chosen for oral presentation and three abstracts were presented orally in both 2009 and 2010 and four were chosen for oral presentation in 2011.

According to Danish law, approval by an ethics committee was not required for this study.

Data will be presented descriptively. Differences between proportions were tested using Fischers test.

## Results

**Figure 1:**
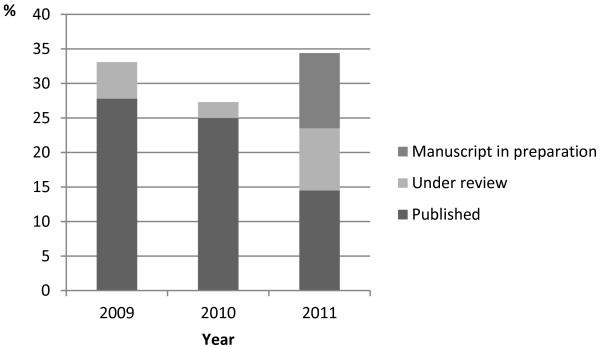
Publication rates 2009–2011.

One hundred nineteen articles were identified that had been presented at the conferences; information regarding publication was obtained for the majority (n = 64) using the e-mail questionnaire while nine additional articles were identified through searching PubMed. No manuscripts were identified as being published in other formats than full-text articles. For two authors a working e-mail address could not be identified and these were excluded from the analysis. The response rate to the questionnaire was 53% after adjustment for non-identification. Figure 1 shows publication rates for 2009–2011 as well as the number of manuscripts under review and preparation (according to authors) at the time of contact (May 2013).

### 2009

Nineteen abstracts were presented at DEMC in 2009. Of these, 6 (31.6%) had been published as full-text articles in peer-reviewed journals. One abstract was under review prior to publication.

### 2010

From the 2010 DEMC, 44 abstracts were published. Eleven (25%) abstracts had been published as full-text articles. One abstract was under review prior to publication.

### 2011

As for the 2011 DEMC, 55 abstracts were published and 8 (14.5%) had been published within 18 months (none of which were oral presentations). Five (9%) were under peer-review (one of which was presented orally), and further six (10.9%) were planned for publication by the authors.

### Oral vs. poster presentation

Ten of the abstracts were presented orally at the conferences, and four of these (40%) had been published, while 21 (19.4%) of the 108 abstracts presented as posters had been published as full-text articles (p = 0.2).

### Impact factor

Full-text articles were published in journals with impact factors ranging from 0.607 to 2.498 (Table 1).

**Table 1:** Impact factor of publishing journals, number of published articles per journal (Type of presentation)

**Journal**	**Impact factor (2012)**	**Number of articles (Type of presentation)**
Acta Cardiol	0.607	1 (poster)
Am J emerg Med	1.704	1 (poster)
BMC Health Serv Res	1.773	1 (poster)
Clin Epidemiol	NA	1 (poster)
Eur J Emerg Med	1.021	3 (2 poster/1 oral)
Int Emerg Nurs	NA	1 (poster)
J of Crit Care	2.498	1 (oral)
Danish Medical Bulletin (Ugeskrift for læger)	0.923	3 (2 poster/1 oral)
J of the Danish Nurses’ Organization (Sygeplejersken)	NA	1 (poster)
Scand J Infect Dis	1.706	1 (poster)
Scand J Gastroenterol	2.156	1 (poster)
Scand J Trauma, Resusc Emerg Med	1.68	4 (3 poster/1 oral)

## Discussion

Approximately one third of the abstracts presented at DEMC 2009 and 2010 were published in peer-reviewed journals by May 2013. For the 2011 DEMC, 14.5% had been published within 18 months after the conference with several articles submitted for peer-review in the same period. When comparing full-text publication rates from DEMC to previous international studies in EM [6,10] it is evident that the Danish EM research community has similar rates of publication. The publication rate is similar to other findings in e.g. cardiology (30.6%) [11] and primary care/family medicine (34.4%) [12].

Presentation of abstracts at scientific meetings is an important activity in knowledge sharing and motivation of researchers, but the criteria for acceptance is not as strict as a peer-review process for full-text articles. Publication of study findings, positive as well as negative, is extremely important as it benefits all interested in a particular field of medical science. However, the issue of positive publication bias is well known, and a previous study [13] demonstrated that it is particularly a concern with conference abstracts, since a subjective “originality” factor, presence of positive results and not the quality of study design is a predictor for acceptance. Hence it can be argued that results from abstracts not published as full-text articles should be interpreted and referred to with caution.

The fact that no manuscripts were identified as being published in other formats (letters, comments etc.) than full-text articles may reflect lack of focus on other ways of publishing results from smaller studies. The reason why app. 70% of presented abstracts is not published is not known. Knowledge of reasons for non-publication could lead to efforts to promote publication like increased funding; the possibility of discussion between authors and editors at conferences; “publication mentors” to ease the writing/submission process; and/or research courses provided by scientific societies.

Our study has limitations. First, we did not examine if certain elements in the content of the abstracts were predictors for later publication as full text articles. Second, a third request for information would most likely have yielded a higher response rate but was not practically feasible.

## Conclusion

Even though emergency medicine (EM) is not recognized as a medical specialty in Denmark, there is a relatively large and active research community as shown by publication rates from the Danish Emergency Medicine Conferences 2009–2011.

Publication rates are comparable to those in EM internationally, however, efforts can be made to clarify reasons for non-publication and initiate activities to promote publication.

## Competing interests

The authors declare that they have no competing interests, however, it should be stated that Dan Brun Petersen is chair of the Danish Society for Emergency Medicine.

## Authors’ contribution

AKR conducted the survey, calculated the statistic, and prepared the manuscript. All authors read and approved the final manuscript.
